# Assimilated Rank

**Published:** 1850-08

**Authors:** 


### THE MEDICAL EXAMINER.

PHILADELPHIA, AUGUST, 1850.

### ASSIMILATED RANK.

On motion of G. C. M. Roberts, M. D., the following preamble and resolutions were unanimously adopted by the "Medical and Chirurgical Faculty of Maryland," at its convention, held June 5, 1850:

*Whereas,* Success in the medical profession requires intelligence, sound morality, and competent knowledge of the principles of medicine, as well as liberal education; and, *Whereas*, humanity and patriotism alike demand that all our fellow citizens who serve the Republic in the Army and Navy should be, when sick or wounded, accompanied by physicians as well instructed as any our country affords; therefore

*Resolved,* That the critical examination of candidates for admission into the Medical Departments of the Army and Navy tends to the improvement of medical education, and to secure competent medical officers in the military service of the country.

*Resolved,* That properly qualified members of the medical profession are *socially* the equals of members of any branch of the Army and Navy, and therefore should be assigned by law a respectable position in every military community.

*Resolved,* That the "Medical and Chirurgical Faculty of Maryland" regards with approbation the law of the United States which confers military rank upon medical officers of the Army, because it secures them an equality of rights and privileges with officers of other staff departments.

*Resolved,* That the "Medical and Chirurgical Faculty of Maryland" earnestly recommend that a similar law be enacted by Congress to place officers of the medical department of the Navy on an equality of rights and privileges with other officers of this branch of the national defence.

*Resolved,* That the Secretary of the Faculty be, and is hereby directed to transmit immediately, copies of these resolutions, properly signed by the officers of the Faculty, to the Secretaries of War and of the Navy, through the chiefs of the medical department of each service at Washington, and to the President of the Senate and Speaker of the House of Representatives of the United States, in order that the attention of Congress may be invited to the subject.

From the minutes.

Wm. H. Davis, Secretary.

*Extract from a pamphlet on "Assimilated Rank in the Navy," by a (presumed) Passed Midshipman.*

"A law of Congress entitled, An Act for the better government of the Navy, approved April 23d, 1800, provides for the distribution of prize money according to the rank of officers in the Navy and Marine corps. Article 1st, provides for the proportion of commanders of fleets and squadrons, and commanders of single ships. Article 2d, provides for sea-lieutenants, captains of marines, and sailing masters. Article 3d, provides for chaplains, lieutenants of marines, surgeons, pursers, boatswains, gunners, carpenters, and master's mates.

"In each of these cases the order in which the different grades are enumerated, and the amount of prize money conceded, is indicative of the rank of the officer specified. Being a law of Congress, fully approved, it is as much a supreme law as any other portion of the existing naval code of which it forms a part, is in full force, and can only be repealed by the passage and approval of another act."

Whether the complement from this association or classification is in favor of the "boatswains, gunners, carpenters, and master's mates," or in favor of "chaplains, lieutenants of marines, surgeons, and pursers," is a question not discussed by our author, who seems to be satisfied that the law is good and sufficient, and that *his* reading is the true one.

